# Polylactol

**Published:** 1914-02-28

**Authors:** 


### Institutional Needs.

### POLYLACTOL.

(The Bayer Co., Ltd., 19 St. Dunstan's Hill, E.C.)

A sample of this new tonic and galactagogue has been submitted to us by the manufacturers, who point out that their well-known preparation, somatose, has long been recognised to possess galactagogue properties and is the basis of polylactol. The further constituents of the latter are iron in organic form, maltose, and galactose. The iron is added for its tonic properties, and the carbohydrates because they are believed to help the carbohydrate and also the fatty constituents of maternal milk. The preparation is palatable and easily taken; the dose is one teaspoonful, dissolved in plenty of milk, given three or four times a day with meals. As a result of clinical experiments, it is claimed that in most cases natural feeding will be possible when polylactol is administered. It is important to observe the recommendations of the manufacturers that the administration should preferably be commenced a week or two before confinement, and continued throughout lactation. A really reliable galactagogue is an article which must always command a large *clientèle* and a ready sale. If the claims put forward on behalf of polylactol are borne out in practice, its success should be assured.

